# It takes two to tango: Concerted protein translation and degradation necessary for synaptic scaling

**DOI:** 10.1371/journal.pbio.3001448

**Published:** 2021-11-24

**Authors:** Ana Luisa Carvalho, Dominique Fernandes

**Affiliations:** 1 CNC—Center for Neuroscience and Cell Biology, University of Coimbra, Coimbra, Portugal; 2 Department of Life Sciences, University of Coimbra, Coimbra, Portugal; 3 Institute of Interdisciplinary Research, University of Coimbra, Coimbra, Portugal

## Abstract

This Primer explores the implications of a new study in PLOS Biology which reveals how homeostatic synaptic plasticity requires coordinated and inter-dependent protein synthesis and degradation, as well as remodeling of the miRNA-induced silencing complex.

## Teaser

Synaptic scaling allows neurons to adjust synaptic strength in response to chronic alterations in neuronal activity. A new study in *PLOS Biology* identifies a pathway that synergizes protein synthesis and degradation with remodeling of the microRNA (miRNA)-induced silencing complex (miRISC) to mediate synaptic scaling.

Activity-dependent synaptic plasticity is thought to be a cellular mechanism for learning and memory. However, if left unchecked, persistent changes in synaptic activity would lead to destabilization of neuronal circuits and function. Homeostatic plasticity mechanisms, such as synaptic scaling, operate at a slower timescale to provide homeostatic control of synaptic activity and maintain neuronal firing rate within a dynamic range [1]. Synaptic scaling has been observed in vitro in cultured neurons subjected to chronic changes in neuronal activity [2,3] and in vivo as a function of development and sensory experience [4,5], and this process is involved in sleep-dependent memory consolidation [6]. The mechanisms at play for the expression of synaptic scaling depend on the proteome remodeling [7] and ultimately result in changes in AMPA-type glutamate receptors at postsynaptic sites. In this issue, Srinivasan and colleagues explore the possibility that protein synthesis and degradation processes work in coordination to control homeostatic synaptic scaling of excitatory synapses [8].

Srinivasan and colleagues describe that concomitant inhibition of the proteasome and translation blocks synaptic downscaling and the decrease in surface AMPA receptors triggered upon prolonged neuronal hyperactivity, suggesting that the 2 processes cooperate to induce synaptic downscaling. Indeed, components of the proteasomal machinery and of the microRNA (miRNA)-induced silencing complex (miRISC) were found to cosediment with translation initiation factors in hippocampal polysomes. This association depends on translating transcripts, since it is disrupted in polysomes isolated from RNAse-treated lysates. Interestingly, prolonged treatment of hippocampal neurons with the GABA_A_ receptor antagonist bicuculline, to induce hyperactivity and consequent synaptic downscaling, led to an enrichment of translation regulators, components of the proteasome, and the miRISC-associated E3 ligase Trim32 in polysomes, but to a depletion of MOV10, a helicase, and RNA-binding protein. This evidence suggests that chronic neuronal hyperactivity impacts on the polysome association of the proteasome and translation machineries, as well as elements of the miRISC.

To understand mechanistically how synaptic downscaling depends on proteomic remodeling that involves both protein translation and degradation, the authors focused on the opposite changes in the expression levels and polysome association of 2 of the miRISC components, Trim32 and MOV10, triggered by chronic neuronal hyperactivity. They found that Trim32 increased expression depends on mTORC-regulated Trim32 translation, which is necessary for proteasome-dependent MOV10 down-regulation. They further showed that Trim32 is the E3 ligase responsible for degradation of MOV10 mediated by the ubiquitin-proteasome pathway.

A critical question addressed by Srinivasan and colleagues is whether the Trim32-MOV10-miRISC axis is sufficient to drive synaptic scaling. The approach used to tackle this issue was 2-fold: (i) MOV10 depletion using RNA interference was found to recapitulate decreased surface AMPA receptor levels and reduced amplitude of AMPA receptor–mediated miniature excitatory postsynaptic currents (mEPSCs), which occluded any further synaptic downscaling induced by prolonged hyperactivity; and (ii) Trim32 knockdown blocked downscaling of mEPSCs triggered by chronic bicuculline exposure. Together, these pieces of evidence support the involvement of the Trim32-MOV10 pathway in synaptic downscaling. Finally, the authors inquired into how this pathway could lead to the removal of surface AMPA receptors to bring about synaptic downscaling. Loss of MOV10 enhanced the expression of the immediate early gene Arc/Arg3.1, which is known to mediate homeostatic synaptic scaling of AMPA receptors via the activation of AMPA receptor endocytosis [9]. In addition, Srinivasan and colleagues observed that bicuculline treatment enhanced the expression of a reporter gene fused with the 3′ UTR of Arc, which was prevented by Trim32 knockdown. On the other hand, MOV10 depletion enhanced the reporter expression under baseline conditions and prevented its further expression upon bicuculline exposure. Thus, hyperactivity-triggered Trim32 translation and consequent MOV10 degradation leads to Arc up-regulation, which may mediate the removal of surface AMPA receptors and thus contribute to synaptic downscaling.

**Fig 1:**
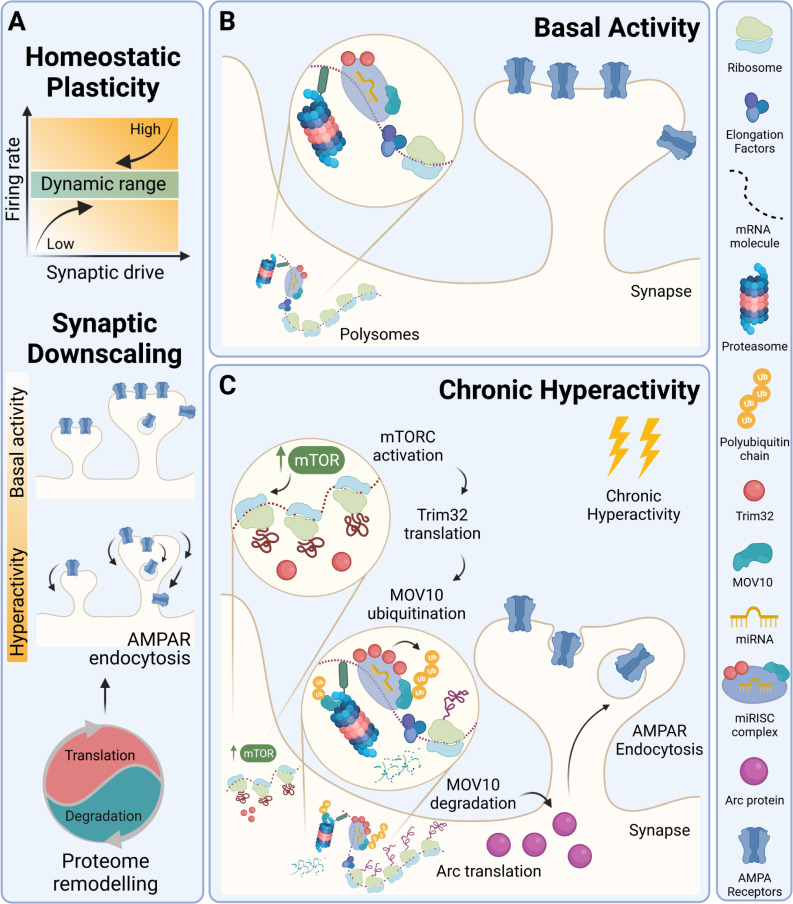
Homeostatic synaptic downscaling requires coupling of protein synthesis and degradation and operates through the Trim32-MOV10 axis in cultured neurons. **(A)** Homeostatic plasticity responses are required to maintain neuronal activity within functional ranges for proper brain function. Upon prolonged neuronal hyperactivity, synaptic downscaling mechanisms operate through the removal of surface AMPA-type glutamate receptors. Proteome remodeling underpins synaptic downscaling and requires concerted protein synthesis and degradation. **(B)** Translating polysomes bring together elements of the translation machinery, components of the proteasome, and of the miRISC in cultured hippocampal neurons. **(C)** Upon prolonged neuronal exposure to hyperactivity, mTORC-regulated Trim32 translation leads to MOV10 polyubiquitination and its proteasome-dependent degradation and to enhanced expression of Arc through posttranscriptional regulation. Through this mechanism, up-regulated Arc levels may mediate the removal of surface AMPA receptors and thus contribute to synaptic downscaling. Created with BioRender.com. miRISC, microRNA-induced silencing complex; miRNA, microRNA.

The study by Srinivasan and colleagues is noteworthy as it supports the compelling idea that proteome dynamics mediating synaptic downscaling requires translation-dependent protein degradation and miRISC remodeling and as it finds that Arc is one of the downstream targets in this pathway, whose expression is regulated at the posttranscriptional level (Fig 1). It is nevertheless possible that other targets relevant for synaptic scaling, in particular those regulated by miRNAs [10], are also under the control of the Trim32-MOV10 axis. This molecular mechanism was described in neurons in which neuronal activity had been manipulated for 24 hours. However, significant changes in the proteome profile also occur in the initial stages of synaptic scaling, and different proteome responses have been described in the neuronal somatic compartment and in processes during synaptic scaling [11]. It remains to be determined in which stage(s) of synaptic downscaling the Trim32-MOV10–dependent mechanism is at play and whether it operates in a compartment-specific or neuron-wide manner. In addition, future studies are required to assess the implication of this newly revealed pathway in experience-dependent homeostatic plasticity in vivo. Given that defects in homeostatic synaptic scaling are associated with the pathogenesis of neurological and neuropsychiatric disorders, the current work sets the stage to investigate whether the disruption of the Trim32-MOV10 pathway can lead to impairment of homeostatic plasticity mechanisms and contribute to circuit and behavioral dysfunctions in disease.

## Abbreviations

mEPSC: miniature excitatory postsynaptic current
miRISC: microRNA-induced silencing complex
miRNA: microRNA
